# Disease burden and high-risk populations for complications in patients with acute respiratory infections: a scoping review

**DOI:** 10.3389/fmed.2024.1325236

**Published:** 2024-05-16

**Authors:** Chendi Cui, Tristan T. Timbrook, Cate Polacek, Zoe Heins, Ning A. Rosenthal

**Affiliations:** ^1^PINC, AI Applied Sciences, Premier Inc., Charlotte, NC, United States; ^2^Global Medical Affairs, bioMérieux, Inc., Salt Lake City, UT, United States; ^3^University of Utah College of Pharmacy, Salt Lake City, UT, United States

**Keywords:** acute respiratory infection, disease burden, high risk, risk factors, epidemiology

## Abstract

**Background:**

Acute respiratory infections (ARIs) represent a significant public health concern in the U.S. This study aimed to describe the disease burden of ARIs and identify U.S. populations at high risk of developing complications.

**Methods:**

This scoping review searched PubMed and EBSCO databases to analyze U.S. studies from 2013 to 2022, focusing on disease burden, complications, and high-risk populations associated with ARIs.

**Results:**

The study included 60 studies and showed that ARI is associated with a significant disease burden and healthcare resource utilization (HRU). In 2019, respiratory infection and tuberculosis caused 339,703 cases per 100,000 people, with most cases being upper respiratory infections and most deaths being lower respiratory infections. ARI is responsible for millions of outpatient visits, especially for influenza and pneumococcal pneumonia, and indirect costs of billions of dollars. ARI is caused by multiple pathogens and poses a significant burden on hospitalizations and outpatient visits. Risk factors for HRU associated with ARI include age, chronic conditions, and socioeconomic factors.

**Conclusion:**

The review underscores the substantial disease burden of ARIs and the influence of age, chronic conditions, and socioeconomic status on developing complications. It highlights the necessity for targeted strategies for high-risk populations and effective pathogen detection to prevent severe complications and reduce HRU.

## 1 Introduction

Acute respiratory infections (ARIs) are categorized as upper respiratory tract infections or lower respiratory tract infections. They can be caused by a wide range of viral and bacterial pathogens, leading to significant morbidity and mortality worldwide (1–3). They account for 4.25 million deaths annually and are a leading cause of death globally (4). Moreover, the burden of ARIs on healthcare systems has been increasingly recognized, with heightened healthcare resource utilization (HRU), hospitalizations, and outpatient visits attributed to these infections (5–7).

In the U.S., ARIs continue to be a major public health concern. The disease burden during the 2021–2022 influenza season was lower than in the pre-pandemic era, potentially due to quarantine measures and other strategies implemented during the COVID-19 pandemic, as well as the observed interruption of care (8). However, the disease burden increased and even surpassed pre-pandemic levels in the 2022–2023 season (8). During the 2021–2022 influenza season, there were an estimated 9 million influenza illnesses, 4 million influenza-related medical visits, 100,000 influenza-related hospitalizations, and 5,000 influenza deaths (9). In contrast, during the 2022–2023 season, there were an estimated 26–50 million influenza illnesses, 12–24 million influenza-related medical visits, 290,000–630,000 influenza-related hospitalizations, and 18,000–55,000 influenza deaths (10). The disease burden also varies by age group, with the highest illness rate among individuals aged 5–17 years, the highest medical visit and hospitalization rate among those aged 0–4 years, and the highest mortality rate in seniors aged 65 or older (9).

While most ARIs resolve within a few weeks with symptomatic relief such as over-the-counter medications, they can also lead to various complications that significantly contribute to their overall disease burden. For instance, complications of influenza include viral pneumonia, bacterial pneumonia, sinusitis, and otitis media (11). The risk of developing complications may vary among different populations (12).

Understanding the disease burden and risk factors associated with an increased risk of developing complications is crucial for clinical practice, public health initiatives, and future research toward targeted interventions. The majority of previous reviews on this topic were published before the COVID-19 pandemic. To gain insights into the epidemiology of ARIs and to identify populations at increased risk of complications in the U.S., this review examines recent literature on the disease burden of ARIs and populations with a high risk of developing complications from these infections.

## 2 Methods

We conducted a scoping literature review and reported according to the Preferred Reporting Items for Systematic Reviews and Meta-Analyses Extension for Scoping Reviews (Supplementary Table S1) (13, 14). The PubMed and EBSCO databases were searched in December 2022 to identify peer-reviewed studies published between January 2013 and December 2022 reporting on disease burden or complications in patients with ARIs. Original research articles, systematic reviews, and meta-analyses were included. Duplicate articles and certain article types (case studies, case reports, clinical trials, studies with sample size less than 100 patients, or studies that demonstrated systematic errors that may affect the validity of results) were excluded. Titles and abstracts of the remaining articles were reviewed, and full-text articles were obtained for further screening. Articles not meeting the inclusion criteria were excluded, as appropriate. Results from the final articles included in this review were categorized into two topics: disease burden of ARI, and populations that have higher risk of developing complications.

### 2.1 Literature search strategy

Studies were searched using key terms related to respiratory tract infections and disease burden or complications (Supplementary Tables S2, S3). Inclusion criteria comprised studies published in the last 10 years, which were either peer-reviewed publications or publications on websites of professional organizations or federal or global agencies. Furthermore, the study populations included those in the U.S., and all selected publications were required to be written in English.

### 2.2 Data extraction

Information extracted from articles included, when available: study type, time period or dates study was conducted; data sources, population size, age range, genders, and race/ethnicity; follow-up period; outcome measurements; key findings and statistics; and (when available) if studies were modeling studies and if population vaccination rates were captured or considered. Following data extraction, two reviewers (C.P., C.C.) discussed analysis of queries on findings. All data extraction was conducted using Microsoft Excel 365 (Microsoft Corporation).

## 3 Results

### 3.1 Included studies

**Figure 1:**
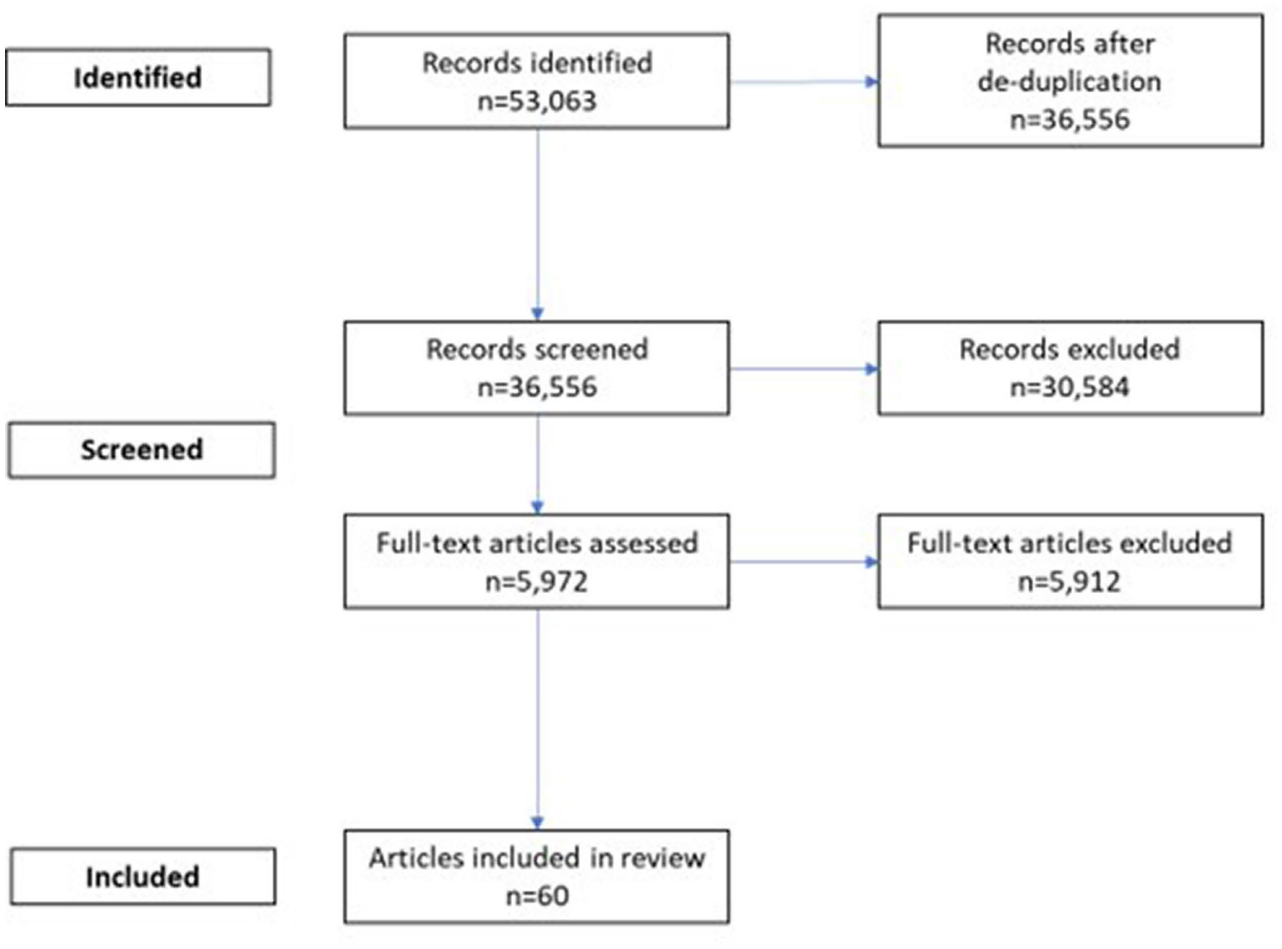
Flow diagram of study selection.

The literature search yielded 5,972 unique records after removing duplicates. After applying exclusion criteria, 60 papers were selected for inclusion in the review, with 39 papers focusing on disease burden (15–54) and 21 papers reporting risk factors associated with the development of ARI complications (Figure 1) (55–74). The findings were summarized in Table 1. Detailed results from each study were included in Supplementary Table S4.

**Table 1:** Summary of findings.

Category	Findings
Pediatric and adult populations at higher risk of developing complications from acute respiratory infections	*Pediatric population* Complications that have been assessed include neurologic complications, pulmonary complications (including pneumonia severity, respiratory failure), multisystem inflammatory syndrome, healthcare resource utilization (HRU) including readmission, intensive care unit (ICU) admission, and length of stay (LOS), and mortality Younger age, male and racial and ethnic minorities (including Asian, Pacific Islander, American Indian, multiracial, and unspecified race/ethnicities) and chronic neurologic conditions were associated with increased risk of neurologic complications Age (older children or infant), comorbidities and chronic conditions (including asthma, congenital respiratory anomalies, congenital musculoskeletal anomalies, chromosomal anomalies, 3+ comorbidities, and complex chronic medical conditions) were associated with increased risk of pulmonary complications Non-Hispanic Black and low social vulnerability index were associated with increased risk of multisystem inflammatory syndrome Age younger than 6 months and chronic conditions (including complex chronic complex conditions, lung disease, cardiovascular disease, and neurologic and neuromuscular disorder) were associated with increased HRU Asthma, comorbidities (including congenital respiratory anomalies, congenital musculoskeletal anomalies, and chromosomal anomalies), and heart and/or lung transplant were associated with increased risk of mortality *Adult population* Complications that have been assessed include intracerebral hemorrhage (ICH), renal complications (including acute kidney injury [AKI] and renal replacement therapy), respiratory complications (including acute respiratory failure, acute respiratory distress syndrome (ARDS), and the use of mechanical ventilation), HRU (including ICU admission, hospitalization, and LOS), and mortality Races other than Caucasian and the use of anticoagulation agents were associated with increased risk of ICH Higher BMI and heart failure were associated with increased risk of renal complications Asthma, higher BMI, heart failure, and malnutrition were associated with increased risk of respiratory complications Older age (≥75 years) and comorbidities including hematological malignancies, chronic kidney disease, chronic heart failure, stroke, previous evidence of pneumonia, and asthma were associated with increased HRU Older age (>65 years), male, alcohol use disorder and alcohol-related complications, higher BMI, malnutrition, heart failure, malignancy with chemotherapy administered and a higher SOFA score were associated with increased risk of mortality *Combined population* One study assessed the association between patient characteristics and ICU admission among patients with influenza A (H1N1) and seasonal influenza Asthma, pregnancy, male, and non-Hispanic were associated with increased risk of ICU admission Individuals younger than 18 years were more likely to have a influenza A (pH1N1)-related ICU stay than those aged 45–64 years Risk for influenza-like illness (ILI)-related ICU stay was greater for individuals aged 5–12 years than for those aged 45–64 years
The disease burden of acute respiratory infections	*Pediatric population* Increases in severe respiratory illness among children and adolescents resulting from enterovirus D68 (EV-D68) infections occurred biennially in the US in 2014, 2016, and 2018, primarily in late summer and fall. EV-D68 levels were lower in 2020, possibly due to COVID-19 mitigation measures (e.g., face masks, hand hygiene, physical distancing) (21) Human metapneumovirus (HMPV) infection is associated with a substantial burden of hospitalizations and outpatient visits among children through the first 5 years of life, especially in the first year (17) RSV is a frequent cause of hospitalization, especially among children aged <2 months (15). In the US population, an estimated 49,509–59,867 community-onset RSV-associated hospitalizations among children aged <2 years occurred during the 2014–2015 season. Among US infants <1 year of age, annual rates of RSV-associated hospitalization ranged from 8.4 to 40.8 per 1000 (22) SARS-CoV-2 affects children of marginalized populations at higher rates than children who come from racial/ethnic majority groups and higher socioeconomic status (20). In one study, Hispanic children represented 46.4% of cases, and Non-Hispanic, Black children represented 30.0% *Adult population* Elderly age is associated with 3–9 times the odds of hospitalization and select comorbidities were associated with 2–7 times the odds of hospitalization Co-morbidities include congestive heart failure, COPD, coronary artery disease, and late-stage chronic kidney disease (46) The increasing elderly population will account for an estimated growth of 45 million people by 2040. If age-specific incidence rates of pneumococcal pneumonia remain stable, outpatient pneumococcal pneumonia visits will likely increase by 43%, and hospitalizations due to pneumococcal pneumonia will increase by close to 100% between 2004 and 2040, with most of the increase occurring in the elderly (53) About 2.2 million outpatient visits for otitis media related to influenza occur annually, of which 86% are in children <18 years (45) Pneumonia as a complication of influenza increases risk of mortality and leads to greater HRU and direct medical costs among patients hospitalized with influenza. These effects are seen early during the index hospitalization and within the first 30 days after diagnosis, but their impact continues throughout a year of follow-up (26) Almost one in five patients who are hospitalized with community-acquired pneumonia (CAP) requires intensive care. Nearly one-half of patients with CAP in the ICU will die within 1 year (28). CAP episodes are also associated with a notable increase in cost during a 90-day CAP episode period, including expenditures related to hospitalization and other inpatient services (39) *Combined population* For all age groups with RSV compared to those without, particularly in the elderly age groups, there are 1.9–3 days length of stay, 0.4–0.5 more ED/urgent care visits, 0.7–2.7 more ambulatory visits, 12.1–18.6 more outpatient visits, and 9.5–14.6 more prescriptions Adjusted mean annual costs between RSV and non-RSV controls is higher in the elderly (≥65; $12,030–$23,194) than in those aged <65 years ($2251–$5391) Among children, adjusted costs attributable to RSV are higher in children aged 5–17 years ($3192), than those 1–4 years ($2251–$2521)

### 3.2 Disease burden of ARI

In 2019, the age-standardized incidence, death, and disability-adjusted life years (DALYs) per 100,000 population of respiratory infection and tuberculosis (RIT) were 339,703 (95% CI 303,184–382,354), 13.6 (95% CI 12.2–14.4), and 384.9 (95% CI 330.6–458.6), respectively (2). Upper respiratory infections constituted the majority of RIT age-standardized incidence rates, while lower respiratory infections made up the highest proportion of RIT age-standardized death and DALY (2).

ARIs significantly contributed to HRU. Approximately 14.5 million outpatient visits for influenza occur annually, with around 80% of visits taking place among the 5–49 age group and approximately 0.7 million visits among seniors aged 65 years and older (45). Each year, an estimated 10% of all children under 18 years and 4% of the entire population < 65 years sought outpatient care for respiratory illness related to influenza (45). Indirect costs, including absenteeism, premature death, and overall direct costs, contributed to the economic burden of influenza, accounting for about $8.0 billion annually in the U.S. (31, 46).

It is noteworthy that the COVID-19 pandemic, which brought about unprecedented changes in societal behavior and public health policies, has had a significant impact on the transmission of non-SARS-CoV-2 respiratory viruses. The adoption of measures such as face masks, improved hand hygiene, social distancing, and the screening and isolation of individuals showing symptoms, along with quarantine protocols, have collectively led to a notable reduction in the community circulation of these viruses (75). Consequently, these changes have been instrumental in substantially decreasing the burden of ARIs in the United States and other parts of the world during the pandemic period (75, 76).

Utilization is likely to continue to increase in the future. The growing elderly population is projected to increase by an estimated 45 million people by 2040, which is expected to significantly contribute to a rise in outpatient pneumococcal pneumonia visits and related hospitalizations (53). Assuming age-specific incidence rates of pneumococcal pneumonia remain constant, outpatient visits for pneumococcal pneumonia are likely to increase by 43%, while hospitalizations due to pneumococcal pneumonia may nearly double between 2004 and 2040 (53). Additionally, ARIs may cause complications that increase HRU. For instance, about 2.2 million outpatient visits occur annually for otitis media related to influenza, with 86% occurring in children under 18 years (45).

Previous research showed each individual ARI pathogen causing significant burden. In recent years, the pediatric population in the U.S. has seen an increase in severe respiratory illnesses due to enterovirus D68 infections, which are most prevalent during late summer and fall (21). Human metapneumovirus infections also pose a significant burden on hospitalizations and outpatient visits for children up to 5 years old, particularly during their first year of life (17). There is substantial annual RSV-attributable HRU and costs in the U.S. across age groups. Among children under 2 years of age, during the 2014–2015 season, it was estimated that between 49,509 and 59,867 community-onset RSV-associated hospitalizations occurred in the U.S., with the highest hospitalization rate in infants under 2 months (15). A previous review showed that the annual RSV-associated hospitalization rates ranged from 8.4 to 40.8 per 1,000 for U.S. infants under 1 year old (22). Across all age groups, RSV infection led to an increased length of hospital stay (1.9–3 days), more emergency department and urgent care visits (0.4–0.5), additional ambulatory visits (0.7–2.7), more outpatient visits (12.1–18.6), and higher number of prescriptions (9.5–14.6) compared to those without RSV, with the highest burden in those aged 65 years and older (42). The adjusted mean annual costs associated with RSV were higher in the elderly population (≥65 years; $12,030–$23,194) than in those under 65 years ($2,251–$5,391) (42). Among children, costs attributable to RSV were higher for those aged 5–17 years ($3,192) than for those aged 1–4 years ($2,251–$2,521) (42).

Various factors may be associated with HRU. Age is one important factor, as increased HRU is associated with both infants under 6 months old and the elderly population (Table 2). Among influenza patients, infants younger than 6 months old were 40% more likely to be admitted to the intensive care unit (ICU) than older infants, and a higher hospitalization rate was observed for infants younger than 3 months old compared to those older than 3 months (64). Elderly age is also linked to 3–9 times the odds of hospitalization (46). One study found that individuals under 18 years of age were more likely to experience an influenza A (H1N1)-related ICU stay than those aged 45–64 years, and the risk for influenza-like illness-related ICU stay was greater for individuals aged 5–12 years than for those aged 45–64 years (74). Additionally, chronic conditions are associated with increased HRU. Specific comorbidities like congestive heart failure, chronic obstructive pulmonary disease, coronary artery disease, and late-stage chronic kidney disease were associated with 2–7 times the odds of hospitalization (46). Increased HRU was also linked to comorbidities like hematological malignancies and chronic kidney disease. ICU admission rate among patients with influenza A (H1N1) or seasonal influenza were higher in patients with asthma or pregnancy (74). Furthermore, socioeconomic factors, such as male gender and non-Hispanic ethnicity, have been found to be associated with influenza A (H1N1)/seasonal influenza-related ICU admission rate (74).

**Table 2:** Age-related findings.

Pathogen/condition	Complications/disease burden	Population	Age-related findings
Community-acquired pneumonia (CAP)	Incidence	Adult	CAP incidence rates were higher for those aged ≥50 years
Influenza A (H1N1)	ICU admission	Combined	Individuals younger than 18 years are more likely to be admitted to the ICU due to influenza A (H1N1) compared to those aged 45–64 years
Influenza A (H1N1)	Mortality	Adult	Adults over the age of 65 are at an increased risk of mortality from influenza A (H1N1)
HMPV	Hospitalization rates	Pediatric	Hospitalization rates for HMPV are highest in infants under 6 months
Influenza	2009 pandemic mortality	Combined	The 2009 influenza pandemic saw a younger age distribution in mortality compared to typical seasonal flu deaths
Influenza	Hospital visits	Combined	~80% of visits occurred in the 5–17 and 18–49 age group
Influenza	Hospitalization	Combined	Elderly age was associated with 9 times the odds of hospitalization (≥65 years vs. 5–17 years) and select comorbidities were associated with 2–3 times the odds of hospitalization
Influenza	Hospitalization rates	Adult	Hospitalization rates for influenza vary among adults, with those aged 50–64 years experiencing different rates
Influenza	Hospitalization rates	Combined	The burden of influenza-associated hospitalizations varies by age, with the highest rates observed in individuals over 65 years
Influenza A (H1N1)	ICU admission	Pediatric	Children under the age of 6 months are at an increased risk of requiring ICU admission due to influenza
Influenza	ICU admission	Combined	Children aged 5–12 years are at a greater risk of requiring ICU admission for influenza compared to older age groups
Influenza	Severe complications	Pediatric	Older children are more likely to experience severe complications from seasonal influenza
Influenza and RSV	Hospitalization	Combined	The highest hospitalization rates for influenza are seen in individuals over 75 years old, while for RSV, infants under 1 year are most affected
Pneumococcal empyema	Incidence	Pediatric	More complicated cases were observed as age increases
Pneumonia	CAP incidence and mortality	Adult	Elderly adults, especially those older than 80 years, experience higher incidence and mortality rates due to CAP
Pneumonia	Hospitalizations, costs	Combined	The elderly population experiences an increased incidence and costs of pneumonia episodes, with projected increases in hospitalizations and associated costs
Pneumonia	Pneumonia severity	Pediatric	1-year-olds and 10-year-olds are at a higher risk of developing severe pneumonia compared to two-year-olds
Respiratory syncytial virus (RSV)	Hospitalization	Adult	Older adults, particularly those over 75 years, are at an increased risk of hospitalization due to RSV
RSV	All-cause mortality	Pediatric	Age was not associated with higher risk
RSV	Costs	Combined	Incremental difference in adjusted mean annual costs between RSV and non-RSV controls was higher in elderly (≥65; $12,030–$23,194) than in those aged <65 years ($2251–$5391). Among children, adjusted costs attributable to RSV were higher in children aged 5–17 years ($3192), than in those 1–4 years ($2251–$2521)
RSV	Hospitalization	Pediatric	Age 0–2 months had highest age-specific RSV hospitalization rates as compared with 3–23 months
RSV	Hospitalization rates, ED visit rates	Pediatric	Hospitalization rates for RSV remain constant over time, with infants aged 0–2 months experiencing higher rates
RSV	Hospitalization rates, Healthcare resource use	Pediatric	Infants under 1 year old have higher hospitalization rates for RSV, with the highest healthcare resource use observed in the elderly population
RSV	Pulmonary complications	Pediatric	Age was associated with higher risk
SARS-Cov-2	Inpatient death, renal replacement therapy, intubation, vasopressors	Adult	Age does not significantly influence these specific complications in adults
SARS-Cov-2	Intracranial hemorrhage (ICH)	Adult	Age is not a significant factor in the occurrence of ICH in adults with COVID-19
SARS-Cov-2	Mortality	Combined	Mortality rates due to COVID-19 vary by age and ethnicity, with some groups experiencing higher rates at different ages
SARS-Cov-2	Neurologic complications	Pediatric	Younger children have a higher likelihood of experiencing neurologic complications

Overall, the results of the studies highlight the significant impact of respiratory infections on healthcare systems, underscoring the need for effective prevention and treatment strategies to mitigate the burden on patients and healthcare resources.

### 3.3 Populations that have higher risk of developing complications

In the pediatric population, complications assessed included neurologic and pulmonary complications, multisystem inflammatory syndrome, and mortality (55–64). In the adult population, complications studied include intracerebral hemorrhage (ICH), renal complications, respiratory complications, and mortality (65–73). The association between older age and complications in adults is also shown in some studies (72, 73).

Studies suggest that younger age may be a risk factor for complications in children. One study of COVID-19 patients demonstrated that the adjusted odds ratio of developing neurologic complications decreased by 3% for each year older in children under age 18 (56). Another study reported that infants younger than 6 months of age had a 40% higher risk of ICU admission compared to older infants with influenza (64). On the other hand, some studies identified a connection between older age and an increased risk of complications in pediatric populations with high-risk conditions, like transplant and cancer patients. For example, one study found that older age correlated with an increased risk of mortality and pulmonary complications in pediatric solid organ transplant patients with respiratory virus infection (57). Similarly, another study observed that older age was linked to an increased likelihood of experiencing severe complications from seasonal influenza in pediatric cancer patients (63). Moreover, one study identified a potential U-shaped relationship between age and the risk of developing severe pneumonia, with higher risk observed in 1- and 10-year-olds than in 2-and 5-year-olds (58), but other the studies did not show this relationship (67, 68). In adults, it has been reported that those over 65 years of age had a 2.1 times higher risk of mortality in cases of influenza A (H1N1) infection (72). Similarly, another study found that patients older than 75 years had an increased risk of hospitalization due to respiratory syncytial virus (RSV) infections, with odds ratios (95% confidence intervals) of 1.73 (1.15, 2.60) for those aged 75–84 compared to those under 65, and 2.53 (1.67, 3.84) for individuals 85 years and older compared to those under 65 (73). However, studies on SARS-Cov-2 have not established a clear association between age and complications (67, 68). Two single-center studies reported that age was not significantly linked to a composite outcome of inpatient mortality, need for renal replacement therapy (RRT), or hemodialysis (HD), intubation, and vasopressor use in patients with COVID-19 (67). Similarly, another study that investigated risk factors for intracranial hemorrhage (ICH) in COVID-19 patients also found no significant association with age (68).

Males have been found to have a 10–90% higher risk of developing complications in patients with influenza (55, 72). However, some studies in patients with COVID-19 did not find an association between sex and complications (62, 67, 68). Regarding race and ethnicity, Asian (55) and non-Hispanic black (62) populations may have a higher risk of developing complications from influenza and COVID-19, respectively, although some studies did not find an association between race, ethnicity, and complications (58, 67). In the pediatric population, the presence of a neurologic chronic condition was associated with a 3 times higher risk of neurological complications in patients with respiratory virus infection (55, 56, 61). Comorbidities and chronic conditions including asthma, malnutrition, congenital anomalies, and complex chronic medical conditions, were associated with an increased risk of developing complications, e.g., ICH, respiratory failure, pneumonia complications, hospitalization, ICU admission, and mortality in patients with influenza, pneumonia, COVID-19, and RSV (58–60, 64, 70, 71, 73). In adults, additional risk factors, such as alcohol use disorder and increased body-mass index, have been associated with mortality in patients with COVID-19 (65, 69).

Overall, studies suggest that age, sex, and chronic conditions are associated with varying risks of complications in both pediatric and adult populations. In children, younger age, especially infants, may generally be a risk factor for complications, while older age is associated with increased risks in high-risk groups, such as transplant and cancer patients. In adults, older age and male sex are associated with higher risks of complications in some studies, although findings are inconsistent across different infections and complications. Additionally, comorbidities and chronic conditions including asthma, malnutrition, congenital anomalies, and complex chronic medical conditions, were associated with an increased risk of complications. Finally, it should be noted that heterogeneity across studies and unadjusted or unmeasured confounding factors may have contributed to the variation in effect seen.

## 4 Discussion

In this study, we conducted a literature review from 2013 to 2022 to examine the disease burden of ARIs and identify populations at a higher risk of developing complications. ARIs are associated with a significant disease burden, consistently imposing considerable strain on HRU and individual health. Factors such as age, chronic conditions, and socioeconomic factors influence HRU and risk of complications. Notably, infants under 6 months, elderly populations, and individuals with comorbidities are more likely to experience increased HRU and complications from ARIs.

Given the substantial impact of ARIs on healthcare systems, early intervention is essential (77). This includes prompt testing, prevention strategies, and vaccination programs, especially for vulnerable populations, such as infants, the elderly, and individuals with chronic conditions (78–80). Early testing is crucial for managing ARIs, as it enables timely identification of pathogens, appropriate treatment, and infection control within communities (81–83). In addition, the recent COVID-19 pandemic highlighted the importance of swift and accurate identification of infectious pathogens to facilitate early treatment and prevent serious disease and complications (84, 85). Public health campaigns should emphasize proper hygiene practices, vaccinations, and early testing when experiencing respiratory infection symptoms.

It’s important to note that even among patients with similar symptoms, the predominant pathogens can vary significantly depending on age and other factors. This was highlighted in a previous study that examined the distribution of common pathogens including human rhinovirus/enterovirus (HRV), adenovirus (Adv), influenza, human metapneumovirus (Hmpv), coronavirus, respiratory syncytial virus (RSV), and parainfluenza (PIV) from under 2 years to 65 years and older (86). Notably, this study found statistically significant differences among all age groups for HRV Adv, influenza, RSV, and PIV. These findings underscore the importance of employing diagnostic methods capable of testing for multiple common pathogens to identify the pathogens.

In addition to hygiene and medical interventions, lifestyle factors like a Mediterranean diet, regular exercise, and healthy sleep habits play a crucial role in reducing infection risks by improving the immune system (87). Preventive measures against chronic diseases, tobacco cessation, moderate alcohol consumption, and maintaining a healthy weight are also vital for immune health (87).

The burden of ARI in the U.S. shows significant variation both seasonally and geographically. Typically, the incidence is highest from November to January and lowest from June to August (88). Geographically, southeastern states, including Florida, Georgia, Alabama, South Carolina, and Mississippi, as well as other south states experience a higher ARI burden compared to states in the North (88). Similar findings were reported by a study which found the highest age-standardized RIT incidence and mortality in the East South Central region (2). Furthermore, the ARI burden is influenced by various factors such as the types of circulating viruses, the effectiveness of available vaccines against these viruses, and the vaccination coverage within the population (89).

Healthcare providers need to be aware of the varying risks of complications in pediatric and adult populations based on factors like age, sex, and chronic conditions. This awareness can inform personalized treatment approaches, potentially improving outcomes and reducing complications. Furthermore, HRU and complication risk differ among various pathogens. Early testing contributes to accurate diagnoses, enabling healthcare providers to initiate appropriate treatments promptly, reducing complication risks and HRU. Policymakers and healthcare administrators should consider the significant burden of ARIs when allocating resources and planning services, ensuring sufficient staffing during peak respiratory infection seasons and investing in diagnostic and treatment facilities, including testing infrastructure.

This study has several limitations. First, our literature search may have missed relevant publications due to the nature of a scoping review. Second, eligible studies may include biases due to study design, definitions, and data sources. Finally, as this was a scoping review, there was no critical appraisal of the included studies or the quality of evidence.

Overall, the findings of this study emphasized the significant disease burden associated with ARIs and highlighted populations with a higher risk of developing complications. The disease burden and complication risks vary by pathogens, suggesting that early detection of pathogens could reduce HRU by applying targeted treatment in the initial phase. Further research is needed in terms of strategies to prevent complications, especially in high-risk populations.

## Author contributions

CC: Investigation, Writing – original draft, Writing – review & editing. TT: Funding acquisition, Investigation, Writing – original draft, Writing – review & editing. CP: Investigation, Project administration, Supervision, Writing – original draft, Writing – review & editing. ZH: Investigation, Writing – original draft, Writing – review & editing. NR: Investigation, Supervision, Writing – original draft, Writing – review & editing.
