# Supplementary material for: Disease burden and high-risk populations for complications in patients with acute respiratory infections: a scoping review
Source: Front Med (Lausanne). 2024 May 16;11:1325236. doi: 10.3389/fmed.2024.1325236 (PMC11138209; doi:10.3389/fmed.2024.1325236)
Supplement: Supplementary file 1 [file Table_1.DOCX]

**Supplementary Materials**

**Table S1.** Preferred Reporting Items for Systematic reviews and Meta-Analyses extension for Scoping Reviews (PRISMA-ScR) Checklist

| **SECTION** | **ITEM** | **PRISMA-ScR CHECKLIST ITEM** | **REPORTED ON PAGE #** |
| --- | --- | --- | --- |
| **TITLE** | | | |
| Title | 1 | Identify the report as a scoping review. | 1 |
| **ABSTRACT** | | | |
| Structured summary | 2 | Provide a structured summary that includes (as applicable): background, objectives, eligibility criteria, sources of evidence, charting methods, results, and conclusions that relate to the review questions and objectives. | 2 |
| **INTRODUCTION** | | | |
| Rationale | 3 | Describe the rationale for the review in the context of what is already known. Explain why the review questions/objectives lend themselves to a scoping review approach. | 3 |
| Objectives | 4 | Provide an explicit statement of the questions and objectives being addressed with reference to their key elements (e.g., population or participants, concepts, and context) or other relevant key elements used to conceptualize the review questions and/or objectives. | 3 |
| **METHODS** | | | |
| Protocol and registration | 5 | Indicate whether a review protocol exists; state if and where it can be accessed (e.g., a Web address); and if available, provide registration information, including the registration number. | N/A |
| Eligibility criteria | 6 | Specify characteristics of the sources of evidence used as eligibility criteria (e.g., years considered, language, and publication status), and provide a rationale. | 4 |
| Information sources* | 7 | Describe all information sources in the search (e.g., databases with dates of coverage and contact with authors to identify additional sources), as well as the date the most recent search was executed. | 4 |
| Search | 8 | Present the full electronic search strategy for at least 1 database, including any limits used, such that it could be repeated. | Supplement table S3 |
| Selection of sources of evidence† | 9 | State the process for selecting sources of evidence (i.e., screening and eligibility) included in the scoping review. | 4 |
| Data charting process‡ | 10 | Describe the methods of charting data from the included sources of evidence (e.g., calibrated forms or forms that have been tested by the team before their use, and whether data charting was done independently or in duplicate) and any processes for obtaining and confirming data from investigators. | 4 |
| Data items | 11 | List and define all variables for which data were sought and any assumptions and simplifications made. | 4 |
| Critical appraisal of individual sources of evidence§ | 12 | If done, provide a rationale for conducting a critical appraisal of included sources of evidence; describe the methods used and how this information was used in any data synthesis (if appropriate). | N/A |
| Synthesis of results | 13 | Describe the methods of handling and summarizing the data that were charted. | 4 |
| **RESULTS** | | | |
| Selection of sources of evidence | 14 | Give numbers of sources of evidence screened, assessed for eligibility, and included in the review, with reasons for exclusions at each stage, ideally using a flow diagram. | 17 |
| Characteristics of sources of evidence | 15 | For each source of evidence, present characteristics for which data were charted and provide the citations. | 4 |
| Critical appraisal within sources of evidence | 16 | If done, present data on critical appraisal of included sources of evidence (see item 12). | N/A |
| Results of individual sources of evidence | 17 | For each included source of evidence, present the relevant data that were charted that relate to the review questions and objectives. | Supplement tables S4 and S5 |
| Synthesis of results | 18 | Summarize and/or present the charting results as they relate to the review questions and objectives. | 5-8 |
| **DISCUSSION** | | | |
| Summary of evidence | 19 | Summarize the main results (including an overview of concepts, themes, and types of evidence available), link to the review questions and objectives, and consider the relevance to key groups. | 8-9 |
| Limitations | 20 | Discuss the limitations of the scoping review process. | 9 |
| Conclusions | 21 | Provide a general interpretation of the results with respect to the review questions and objectives, as well as potential implications and/or next steps. | 9 |
| **FUNDING** | | | |
| Funding | 22 | Describe sources of funding for the included sources of evidence, as well as sources of funding for the scoping review. Describe the role of the funders of the scoping review. | 9 |

**Table S2.** Search strategy key terms

| **CONCEPT** | **SEARCH TERMS** |
| --- | --- |
| Respiratory tract infection disease | (respiratory tract infection* OR respiratory syncytial virus OR influenza OR coronavirus OR metapneumovirus OR rhinovirus OR enterovirus OR pneumonia [OR respiratory viral infection |
| Complication | (complication*) |
| Disease burden | (burden OR incidence OR prevalence) |
| Dates | 2013:2022 |
| Language | eng |

**Table S3. Search strategy for PubMed**

***Pediatric and adult populations at higher risk of developing complications from acute respiratory infections***

| # | Search String | Results |
| --- | --- | --- |
| 1 | (respiratory tract infection* OR respiratory syncytial virus OR influenza OR coronavirus OR metapneumovirus OR rhinovirus OR enterovirus OR pneumonia OR respiratory viral infection*) [tiab] | 396,889 |
| 2 | AND (complication*) [tiab] | 33,404 |
| 3 | AND (pediatric OR adult OR children) [tiab] | 6,694 |
| 4 | AND (risk) [tiab] | 2,375 |
| 5 | Exclude older than 10 years, non-English, non-human studies | 1,103 |

***Disease burden of acute respiratory infections***

| # | Search String | Results |
| --- | --- | --- |
| 1 | (respiratory tract infection* OR respiratory syncytial virus OR influenza OR coronavirus OR metapneumovirus OR rhinovirus OR enterovirus OR pneumonia OR respiratory viral infection*) [tiab] | 396,889 |
| 2 | AND (burden OR incidence OR prevalence) | 50,688 |
| 3 | Exclude older than 10 years, non-English, non-human studies | 4,830 |

**Table S4. Pediatric and adult populations at higher risk of developing complications from acute respiratory infections**

| Reference | Date range studied | Methodology | Extracted key findings |
| --- | --- | --- | --- |
| **Pediatric population** | | | |
| Antoon 2021^1^ | 2015-2020 | - Cross-sectional study - Assessed length of stay (LOS), intensive care unit (ICU) admission, ICU LOS, 30-day hospital readmissions, deaths, and hospital costs associated with these events. - Patient-level risk factors associated with neurologic complications were identified using multivariable logistic regression | - Influenza and neurologic complications   - Male, Asian and other race (which includes Pacific Islander, American Indian, multiracial, and other unspecified race/ethnicities), and chronic neurologic conditions were associated with higher risk. |
| Antoon 2022^2^ | 2020 to 2022 | - Assessed length of stay (LOS), ICU admission, 30 day readmissions, deaths, and hospital costs. - Used multivariable logistic regression to identify factors associated with neurologic complications. | - SARS-Cov-2 and neurologic complications   - Younger age and chronic neurologic conditions were associated with higher odds. |
| Danziger-Isakov 2019^3^ | 2010 to 2013 | - Retrospective observational cohort chart review - Incidence of RVI was calculated, and the association of baseline SOT factors with subsequent pulmonary complications and death was assessed | - Respiratory virus infection and all-cause mortality   - Heart/lung/heart-lung transplant were associated with higher risk.   - Age, respiratory virus infection onset within 60 days of solid organ transplant, respiratory support at baseline, previous chronic lung disease, and received intravenous immunoglobulin within 2 weeks were not associated with mortality. - Respiratory virus infection and pulmonary complications   - Age was associated with higher risk.   - Heart/lung/heart-lung transplant, respiratory virus infection onset within 60 days of solid organ transplant, respiratory support at baseline, previous chronic lung disease, and received intravenous immunoglobulin within 2 weeks were not associated with pulmonary complications. |
| Williams 2016^4^ | 2010 to June 2012 | - Developed risk models to predict severe pneumonia outcomes - In-hospital outcomes were organized into an ordinal severity scale outcomes. Predictors were evaluated in models, and ordinal regression was used for model development. Predictive accuracy was estimated by using discrimination (concordance index). | - Pneumonia and pneumonia severity   - Compared to 2-yr-old, 1-yr-old and 10-yr-old had higher risk of developing severe pneumonia.   - Having 3+ comorbidities was associated with increased risk of severe pneumonia.   - Race/ethnicity household smoke exposure and season were not associated with severe pneumonia. |
| Ghimire 2020^5^ | data from in-hospital pediatric patients from 2003, 2006, 2009, 2012 and 2016 using the  nationally representative Kids Inpatient Database (KID). | - Used weighted data to compare incidence of in-hospital mortality and rates of complications | - Influenza and in-hospital mortality   - Asthma is associated with lower risk of in-hospital mortality.   - Congenital respiratory anomalies, congenital musculoskeletal anomalies, and chromosomal anomalies were associated with increased risk of in-hospital mortality. - Influenza and respiratory failure   - Asthma, congenital respiratory anomalies, congenital musculoskeletal anomalies, and chromosomal anomalies were associated with increased risk of respiratory failure. |
| Leyenaar 2014^6^ | 2007 and June 30, 2010 | - Differences in disease management and outcomes were assessed using multivariable regression | - - Pneumonia and pneumonia complications   - Complex chronic conditions were associated with increased odds of pneumonia complications.   - Pneumonia and 30-day readmission   - Complex chronic conditions were associated with increased odds of 30-day readmission.   - Pneumonia and LOS   - Complex chronic conditions were associated longer LOS. |
| Wilking 2014^7^ | 2009 and June 2010 | - Retrospective descriptive study - Clinical characteristics and outcomes were collected and described in relation to patients’ CNS disease. | - Influenza A(H1N1) and central nervous system manifestations   - Neurological pre-existing conditions were associated with increased risk. |
| Zambrano 2022^8^ | 2020 | - Case-control study - Evaluated race, ethnicity, social vulnerability index (SVI), insurance status, weight-for-age and underlying medical conditions as risk factors using mixed effects multivariable logistic regression. | - SARS-Cov-2 and multisystem inflammatory syndrome   - Non-Hispanic Black and low social vulnerability index were associated with increased risk.   - Children who were previously healthy had higher risk.   - Sex, other race/ethnicity, health insurance, and respiratory system disorder were not associated with the outcome. |
| Carr 2013^9^ | 2002-2009 | - Abstracted demographic data and clinical findings from the time of onset of symptoms of influenza until death or the complete resolution of acute illness | - Seasonal influenza and severe complications   - Older age, recipients of hematopoietic stem cell transplantation, neutropenic patients, and concurrent infections were associated with increased risk. |
| Chaves 2014^10^ | 2003 to 2012 | - Used population-based, laboratory-confirmed influenza hospitalization surveillance data to describe the impact of influenza by age category. - Logistic regression was used to explore risk factors for intensive care unit (ICU) admission. - Adjusted age-specific, influenza-associated hospitalization rates were calculated and applied to the number of US infants to estimate national numbers of hospitalizations | - Influenza and ICU admission   - <6 months and comorbidities including lung disease, cardiovascular disease, and neurologic and neuromuscular disorder were associated with increased risk of ICU admission. |
| **Adult population** | | | |
| Bailey 2022^11^ | Not specified | - Retrospective cohort study - Frequencies and percentages or medians and interquartile ranges of all demographic, clinical, and outcome measures within each of these cohorts were calculated. - Comparisons of measures between those with and without AUD were made using chi-squared tests or Wilcoxon rank-sum tests. - A multiple logistic regression model was used to evaluate the risk of hospitalization between those with and without AUD. - A similar logistic regression model was used to compare all-cause mortality rates between those with and without AUD, and then limited to those in the hospitalized cohort. | - SARS-Cov-2 and all-cause mortality   - Alcohol use disorder or alcohol-related complications were associated with higher odds of all-cause mortality. |
| Hussein 2020^12^ | 2020 | - Multi-center retrospective study - Comparison between the asthmatic and non-asthmatic cohorts was performed. - Continuous variables were described as medians and interquartile ranges, while categorical data were presented as frequencies and percentages. Chi-square or Fisher’s Exact tests were used for categorical variables. Student’s t and Mann-Whitney U tests were applied for quantitative variables to examine the difference between asthmatic and non-asthmatics groups. Shapiro-Wilk test was used to test the normality of the continuous variables. Kaplan-Meier survival analysis and the Log Rank test was used to compare the in-hospital mortality in the two groups. Binary logistic regression analysis was used to assess the role of asthma comorbidity in the outcomes of COVID-19 disease. Age, gender, and obesity were adjusted in the model. Hosmer-Lemeshow test was used to assess goodness-of-fit. | - SARS-Cov-2 and intubation   - Asthma was associated with increased risk of intubation. - SARS-Cov-2 and extubation   - Asthma was associated with increased risk of intubation. - SARS-Cov-2 and total LOS   - Asthma was associated with longer LOS. - SARS-Cov-2 and other procedures (re-intubation, mechanical ventilation, ICU admission), days of events (intubation days, ventilation days, ICU LOS, ICU-free days, time to death), complications (ARDS, renal failure/AKI, sepsis, bacteremia), current state (still hospitalized), mortality   - Asthma was not associated with any of the above. |
| Fox 2021^13^ | 2020 | - Single-center, retrospective observational study - Demographic variables were presented using descriptive statistics and frequencies. Categorical variables were analyzed with chi-square testing. Demographic and clinical variables were tabulated. Independent t-test was used for continuous variables. For skewed variables, Mann–Whitney U test was used to compare differences. Outcomes such as inpatient death, need for renal replacement therapy or hemodialysis (RRT/HD) and need for vasopressors or intubation were considered. Multivariate logistic regression was used to evaluate the factors associated with mortality among patients with diabetes and COVID-19. | - SARS-Cov-2 and inpatient death, need for renal replacement therapy/hemodialysis, intubation and vasopressors   - Age, BMI, sex, race/ethnicity, chronic obstructive pulmonary disease (COPD), asthma, heart failure, cardiovascular artery disease, hypertension, atrial fibrillation, chronic kidney disease, and diabetes were not associated with any of the above. |
| Melmed 2021^14^ | 2020 | - Retrospective cohort study - Used regression models to estimate odds ratios and 95% confidence intervals of association between ICH and covariates. - Used regression models to determine association between ICH and mortality. | - SARS-Cov-2 and ICH   - Races other than Caucasian and the use of anticoagulation were associated with increased risk of ICH.   - Age, male, and hypertension were not associated with ICH. |
| Page-Wilson 2021^15^ | 2020 | - Retrospective cohort study - Manual chart review survey - Data expressed as frequencies and percentages for categorical variables and were tested either by the Chi-Squared test or Fisher’s Exact test depending on size (>5). Continuous variables expressed as either mean (SD) or median (IQR) depending on normality which was tested via QQ plots, and compared using t-test or Mann-Whitney test respectively in bivariate analyses and ANOVA or Kruskall-Wallis in comparisons with more than two groups. Post-hoc pairwise comparisons of outcomes across BMI groups used Bonferroni to correct for multiple comparisons. | - SARS-Cov-2 and mortality   - Increased BMI was associated with increased risk of mortality. - SARS-Cov-2 and intubation   - Increased BMI was associated with increased risk of intubation. - SARS-Cov-2 and septic shock   - Increased BMI was associated with increased risk of septic shock. - SARS-Cov-2 and renal replacement therapy   - Increased BMI was associated with increased risk of renal replacement therapy. - SARS-Cov-2 and ICU admission   - BMI was not associated with ICU admission. - SARS-Cov-2 and ICU time   - BMI was not associated with ICU time. |
| Panhwar 2019^16^ | 2013-2014 | - Used propensity score matching to match patients across demographics, discharge weights, and comorbidities. | - Influenza and in-hospital mortality   - Heart failure is associated with increased risk. - Influenza and AKI   - Heart failure is associated with increased risk. - Influenza and AKI requiring dialysis   - Heart failure is associated with increased risk. - Influenza and acute respiratory failure   - Heart failure is associated with increased risk. - Influenza and acute respiratory failure requiring mechanical ventilation   - Heart failure is associated with increased risk. - Influenza and LOS   - Heart failure is associated with longer LOS. |
| Ponce 2022^17^ | 2018-2021 | - Used multivariable logistic regression to determine whether malnutrition was associated with mortality and adverse events. | - SARS-Cov-2 and death or transfer to hospice, invasive mechanical ventilation, ARDS, extracorporeal membrane oxygenation, and hospital-acquired pressure injury   - History of malnutrition was associated with increased risk of all of the above.   - Hospital-acquired malnutrition was associated with increased risk of all of the above. |
| Shah 2015^18^ | 2013-2014 | - Retrospective cohort study - Descriptive statistics tabulated for demographic data, symptoms, comorbidities, length of stay, laboratory test results, radiologic studies, complications, treatment, SOFA score and support measures. An epidemic curve was generated. The case fatality rate was calculated. - Bivariable analyses compared potential risk factors for death < 30 days after ICU admission | - Influenza A(H1N1) and mortality   - Older age (>65 years), male, history of malignancy with chemotherapy administered within the prior six months and a higher SOFA score were associated with increased risk. |
| Wyffels 2020^19^ | 2011-2015 | - Logistic regression models were developed to determine predictors of initial hospitalization. - Healthcare utilization and costs for 180 days pre- and post-RSV diagnosis were compared. | - Respiratory syncytial virus (RSV) and hospitalization   - Older age (≥75 years), comorbidities such as hematological malignancies, chronic kidney disease, chronic heart failure, stroke, and previous evidence of pneumonia were associated with increased risk. |
| **Combined population** | | | |
| Placzek 2014^20^ | 2008-2009 | - Calculated Diagnostic Cost Group (DxCG) risk score as a measure of comorbidity. - Used logistic regression predictive models to compare ICU stay predictors. | - Influenza A(H1N1) and ICU admission - Individuals younger than 5 years, 5 to 12 years, and 13 to 18 years were more likely to have a influenza A(pH1N1)-related ICU stay than those aged 45 to 64 years. - Females and Hispanics were at less risk for ICU stay. - Within the influenza A(pH1N1) cohort, asthma was highly predictive of ICU admission among those younger than 5 years, 5 to 12 years, and 13 to 18 years. - Pregnancy was also strongly associated with ICU admission among those aged 26 to 44 years. - Seasonal influenza and ICU admission - Risk for ILI-related ICU stay was greater for individuals aged 5 to 12 years than for those aged 45 to 64 years. - Stratified by age group, asthma is a predictor of ILI-related ICU stay among those younger than 5 years. - Obesity was shown to be protective against influenza-associated ICU stay among those aged 45 to 64 years. |

**Table S5. Disease burden of acute respiratory infections**

| Reference | Date range studied | Methodology | Extracted key findings |
| --- | --- | --- | --- |
| **Pediatric population** | | | |
| Arriola 2019^21^ | 2014-2015 | - Used surveillance populations to estimate age-specific rates of RSV-associated hospitalization, after adjusting for detection probabilities. | - 27% admitted to ICU, 6% needed mechanical ventilation, 5 died. - Most cases (1047/1554; 67%) had no underlying condition. - Adjusted age-specific RSV hospitalization rates per 100 000 population were 1970, 897, 531, and 358 for ages 0–2, 3–5, 6–11, and 12–23 months, respectively. - Extrapolating to the larger US population, an estimated 49 509–59 867 community-onset RSV-associated hospitalizations among children aged <2 years occurred during the 2014–2015 season. |
| Chirikov 2020^22^ | 2004-2015 | - Infants with RSV infection in the first year of life were matched to controls and remaining imbalances in the number of individuals in each group were adjusted using propensity score methods. - All-cause, respiratory-related, and asthma/   wheezing-related 5-year average cumulative costs were measured. | - Early premature (n = 213), premature (n = 397), late premature (n = 4446), and full-term (n = 33 417) RSV-infected infants matched to 424, 791, 8875, and 66 735 controls, respectively. - After 2 years from RSV diagnosis, all-cause cumulative costs for RSV-infected infants compared to those for controls increased by $22 081 for early premature infants, by $14 034 for premature infants, by $10 164 for late premature infants, and by $5404 for full-term infants. - 5-year RSV burden increased to $39 490, $23 160, $13 755, and $6631, respectively. - RSV burden was higher when stratified by inpatient and outpatient setting and respiratory-related and asthma/wheezing-related costs. |
| Edwards 2013^23^ | 2003-2009 | - Prospective, population-based surveillance - Prospectively collected demographic and clinical data and compared with Pearson chi-square test or Wilcoxon rank-sum test, as appropriate for categorical or continuous variables. - Used logistic regression to compare between inpatient and outpatient settings among HMPV-positive or HMPV-negative children, with adjustment for age as a continuous variable for each potential risk factor. | - HMPV detected in 200 of 3490 hospitalized children (6%), 222 of 3257 children in outpatient clinics (7%), 224 of 3001 children in the emergency department (ED) (7%), and 10 of 770 asymptomatic controls (1%). - Overall annual rates of hospitalization associated with HMPV infection were 1 per 1000 children <5 years of age, 3 per 1000 infants <6 months of age, and 2 per 1000 children 6 to 11 months of age. - Children hospitalized with HMPV infection were older and more likely to receive a diagnosis of pneumonia or asthma, to require supplemental oxygen, and to have a longer ICU stay. - Estimated annual burden of outpatient visits associated with HMPV infection was 55 clinic visits and 13 ED visits per 1000 children. |
| Fell 2017^24^ | 2000-2014 | - Systematic review | - Reported incidence of lab-confirmed influenza (LCI) hospitalization ranged from 9.3 to 91.2 per 10 000 infants under 6 months for seasonal influenza. - US-based estimate for pandemic influenza A(H1N1) influenza was 20.2 per 10 000 infants. - Rate of LCI illness based on outpatient clinic visits among infants under 6 months was 2.8 per 100 infants in 2002–2003 and 5.9 per 100 infants in 2003–2004. - Estimated rates of LCI hospitalization of infants less than 6 months of age during seasonal epidemics varied from a low of 9.3 per 10 000 infants in 2006–2007 to a high of 91.2 per 10 000 infants in 2003–2004. - Estimate for the 2009 pandemic influenza A(H1N1) time period was 20.2 per 10 000 infants. |
| Fletcher 2014^25^ | 1990–2012 | - Literature review | - Pneumococcal empyema incidence increased among children aged 2–4 years from 1.1 to 2.5 per 100,000 population from 1996-1998 to 2005–2007, respectively - Among children with pneumococcal pneumonia, the proportion of cases with complicated pneumococcal pneumonia increased with age from 26.4% (ages 0–12 months) to 53.0% (ages >61 months) |
| Kurup 2022^26^ | 2020-2021 | - Literature review | - Among positive cases, Hispanic children represented 46.4% of cases and Non-Hispanic, Black children represented 30.0% of cases. - Non-Hispanic, White children represented 7.3% of positive cases. |
| Ma 2022^27^ | 2018-2022 | - Analyzed surveillance data to characterize reported trends in acute respiratory illness, asthma/reactive airway disease exacerba­tion, and percentage of positive RV/EV and EV-D68 test results | - Increases in severe respiratory illness and acute flaccid myelitis (AFM) among children and adolescents resulting from enterovirus D68 (EV-D68) infections occurred biennially in the United States during 2014, 2016, and 2018, primarily in late summer and fall. - EV-D68 levels were lower than expected in 2020, potentially because of implementation of COVID-19 mitigation measures (e.g., wearing face masks, enhanced hand hygiene, and physical distancing). - In August 2022, clinicians in several geographic areas noted an increase in hospitalizations of pediatric patients with severe respiratory illness and positive rhinovirus/enterovirus (RV/EV) test results. - Surveillance data demonstrated an increase in ED visits for children and adolescents with ARI and asthma in late summer 2022 and an increase in percentage of positive RV/EV test results in national laboratory-based surveillance and the percentage of positive EV-D68 test results in pediatric sentinel surveillance. |
| McLaughlin 2020^28^ | 2000-2019 | - Systematic review and meta-analysis | - Among US infants <1 year of age, annual rates of RSV-associated hospitalization ranged from 8.4 to 40.8 per 1000 with a pooled rate of 19.4. |
| Simoes 2022^29^ | 2000-2009 | - Extrapolated data collected from sentinel sites | - RSV hospitalization causes 9% to 12% of all nonbirth hospitalizations and results in 80,000 to 100,000 hospitalizations annually and almost 3 times that number of visits to the ED. - Medicaid-insured infants have approximately 2 times the rate of hospitalization of those with private payers, accounting for 62% of RSV hospitalizations and 56% of ED visits. - Of all children hospitalized, between 15% and 20% go to the ICU and approximately half receive mechanical ventilation - Estimated overall burden of medically attended RSV lower respiratory tract infection (LRTI) in infants varies between 50 and 180/1000, 80% of which occurs in full-term infants. - Average cost of RSV hospitalization is approximately $12 000 - Medicaid pays approximately 2 of 3 of what private payers pay to hospitals and healthcare systems, because of the high rate of hospitalization, Medicaid covers approximately 61% of the aggregate $472 million average cost of hospitalizations. - Infants on Medicaid account for a disproportionate portion (62%) of the RSV deaths. |
| Suh 2022^30^ | 2000-2021 | - Systematic review | - Annual average RSV hospitalization rates (range, 11.6 per 1000 per year among infants aged 6–11 months in 2006 to 50.1 per 1000 per year among infants aged 0–2 months in 1997). - RSV hospitalization rates by primary diagnosis (range, 22.0–22.7 per 1000 in 1997–1999 and 1997–2000, respectively). - RSV hospitalization infants with high-risk comorbidities had 5-times more mechanical ventilation use compared to non–high-risk infants in 1997-2012. |
| Suh 2022^31^ | 2011–2019 | - Use weighted numbers and proportions of hospitalizations or ED visits to identify subgroups carrying greatest weight of morbidity due to disease. - Rates expressed per 1000 live births and described risk of hospitalization or ED visit for each subgroup to identify subgroups bearing greatest risk of morbidity due to disease | - Average annual RSV hospitalization and RSV ED visits were 56 927 (range, 43 845–66 155) and 131 999 (range, 89 809–177 680), respectively. - RSV hospitalization rates remained constant over time, whereas ED visit rates increased. - From 2011 through 2019, Medicaid infants had the highest average rates (RSV hospitalization: 22.3 per 1000; ED visits: 55.9 per 1000) compared to infants with private or other/unknown insurance. - From 2011 through 2019, for all races and ethnicities, Medicaid infants had higher average RSV hospitalization rates (up to 7 times) compared to infants with private or other/unknown insurance. - RSV hospitalization mortality remained constant over time, whereas mechanical ventilation use (2019: 13% of RSV hospitalization) and mean charge during hospitalization (2019: $21 513) increased. |
| **Adult population** | | | |
| Bolge 2021^32^ | 2007–2020 | - Real-world retrospective data - Final 1:1 matching determined using propensity score matching | - During index hospitalization, cases vs. controls had longer LOS [mean (SD): 6.5 (8.3) vs. 1.9 (3.7)], greater ICU use (38.4 vs. 16.8%), and greater mechanical ventilation use (invasive: 11.4 vs. 2.3%; non-invasive: 6.8 vs. 2.6%). - Cases had higher readmission rates than controls (12.3 vs. 3.5% within 30 days; 20.0 vs. 6.1% within 90 days). - Post-index date direct all-cause healthcare costs were higher for cases than for controls (median total cost: $18,428 vs. $621 for 30 days; $21,774 vs. $3312 for 90 days; $25,960 vs. $8699 for 6 months; $35,875 vs. $21,619 for 1 year). |
| Brown 2018^33^ | 2014 to 2015 | - Retrospective analysis - Hospitalizations for each condition were described by length of stay, readmissions, mortality, and total costs. | - 16,430 CAP hospitalizations during 2015 – a rate of 846.7 hospitalizations per 100,000 person-years - Mean [SD] LOS for CAP hospitalizations was 5.2 [6.2] days - 30-day readmission rate percent was 10.0% - Mortality rate per 100,000 persons was 22.5 - Cost per index hospitalization was $13,825 - Total cost of index CAP hospitalizations was $227.1 million - Total readmission costs associated with CAP were $23.3 million - Total costs, including sum of index hospitalization costs and associated readmission, totaled more than $250.4 million for CAP |
| Cavallazzi 2020^34^ | 2014-2016 | - Secondary analysis of a prospective population-based cohort study | - Incidence of CAP in the ICU was 145 cases per 100,000 population of adults. - Cases were clustered in patients from areas with high poverty. - Mortality rate of patients was 27% at 30 days and 47% at one year. - Estimated number of patients hospitalized with CAP requiring ICU was 356,326 per year, and estimated number of deaths at 30 days and one year were 96,206 and 167,474, respectively. |
| Charu 2013^35^ | 1990-2010 | - Calculated influenza-attributable excess deaths by season based on Poisson regression models driven by indicators of respiratory virus activity, seasonality, and temporal trends. | - Mortality burden of the 2009–10 pandemic was ~0.7 times that of a typical influenza season in the past decade and ~0.4 times that of the severe 2003–04 epidemic. - Excess mortality estimates extrapolated from inpatient deaths were within 11% of estimates based on vital statistics for seasonal influenza and 17% for the truncated April–December 2009 pandemic season. - ~85% of excess deaths occur in persons >65 years - During the 2009–10 pandemic, age distribution of inpatient excess deaths differed from previous seasons - greater proportion of deaths occurring among persons aged 5–64 years, especially for broader outcomes - For 5- to-64-year-olds, pandemic mortality rates were 3–10 times those of a typical influenza season and 1–4 times those during the severe 2003/04 epidemic, depending on outcome. In contrast, the pandemic burden in individuals over 65 years was three- to seven-fold lower than in a typical season - There were 14,800 excess respiratory and cardiac deaths to influenza A(H1N1) circulation during April 2009–April 2010 - Estimated 474,000 years of life lost (YLL) attributable to the pandemic based on R&C causes, similar to YLL burden of a typical season, and is ~2/3 that of the severe 2003/04 epidemic season |
| Datta 2017^36^ | 2015-2016 | - Retrospective medical record and laboratory review - Tested equivalence of viral testing rates during periods of peak RSV and influenza activity - Large sample Z-test based on Poisson distributional assumptions used to compare rates of tests performed per ED and hospital admission for respiratory related illness. | - Of adult ED and inpatient tests performed, 136 were positive for RSV, 417 for influenza. - Most adult RSV detections were in hospitalized patients (110 admissions vs 26 seen in ED and discharged), whereas influenza cases were more evenly split (188 admissions vs 218 seen in ED and discharged). |
| De Courville 2022^37^ | 2007-2020 | - Systematic literature review of studies reporting primary influenza-related cost data (direct or indirect) or absenteeism data | - Overall direct costs ranged from $161 to $363 per medically attended case. - Higher overall direct costs for patients visiting an ED or hospitalized - Overall direct cost per medically attended case was 2.1 to 2.7 times higher in complicated cases compared to uncomplicated cases - Hospitalization costs accounted for 73 to 75% of overall direct costs of influenza in adult populations aged 15 or 18 to 64 years - Cost per hospitalization ranged from US$7067 among all adult patients with uncomplicated influenza to US$38,662 among all 45- to 59-year-olds - Cost of influenza-attributable hospitalization is up to 2.5 times higher in an at-risk population versus a not at-risk population. - Indirect costs were consistently higher for patients aged 50 to 64 than for patients aged 18 to 49. - Higher absenteeism costs in Veteran populations with underlying chronic conditions (diabetes, asthma, chronic cardiovascular, or lung disease) compared with the general population - the proportion of patients taking sick leave increased with age within the 18- to 64-year-old age group and for at-risk populations compared with a general population - Average duration of sick leave among at-risk populations was up to 1.8 times higher compared with the general population. - In the general population, costs continued to increase for those aged ≥65, but in US veterans, total economic burden was lower in those aged ≥65 than those 18 to 49 or 50 to 64. |
| Drijkoningen 2013^38^ | 1999-2013 | - Review | - Incidence rates for CAP requiring hospitalization were estimated to be 2.7 per 1000 - In nearly16 000 invasive pneumococcal disease (IPD) cases in adults, infection origin was pneumonia in 53%. - IPD accounted for over 36 000 cases in 2011. - Incidence of IPD was strongly age-related - 38% of cases occurring in children under 2 years of age and another 54% in adults above 50 years of age. - IPD was more common in blacks than in whites (incidence rates 16.6 vs 11.0/100 000) - Hospitalization rates for elderly patients with pneumonia tended to increase - 20% increase between 1988–1990 and 2000–2002. - Among adults aged 50 years or older, nearly 30 000 cases of IPD and more than 500 000 cases of non-bacteremic pneumococcal pneumonia were estimated to occur yearly, resulting in more than 25 000 pneumococcus-related deaths - Direct healthcare costs of pneumococcal disease totaled $3.5 billion in 2004. Pneumococcal pneumonia accounted for 72% of these costs - Hospital costs from CAP were estimated to be $7000 to $8000 per episode. |
| Ehrlich 2021^39^ | 2020 | - Number of new cases in nursing home on calendar day offset by person-days at risk on day was modeled as a Poisson regression | - Before the first point prevalence survey (PPS), nursing homes had an average of 36 COVID-19 cases (27.7% infected; range 0–81 cases, 0%–86.1% infected). - 601 cases detected in facilities during the first PPS. - After initial round of PPS, 44 resident cases were identified in all subsequent rounds of PPS testing, of which 9 (20.4%) were symptomatic at the time of testing - The second PPS identified 20 cases (n = 34 nursing homes), and subsequent PPSs identified an additional 8 (n = 33), 6 (n = 31), 4 (n = 28), 3 (n = 25), 2 (n = 22), 0 (n = 18), 0 (n = 9), 0 (n = 4), and 0 (n = 1) cases in residents - In between PPSs, 93 additional resident cases were also detected, of which 70 (75.3%) were symptomatic at the time of testing. Most (85, 90.3%) cases were identified during the longer period between the first and second round of PPS testing. More than half (60.2%) of cases were detected within 1 incubation period following the first PPS, when exposure in those persons had likely already occurred. - Two nursing homes contained most cases, reporting 38 and 20 cases in the 44 days between their first and second PPS |
| El Chaer 2017^40^ | 2012-2015 | - Retrospective study of all lab-confirmed hMPV infections - Clinical characteristics, risk factors for progression to an LRI, treatment, and outcomes in patients with cancer were determined | - 181 HMPV infections identified in 90 patients (50%) with hematologic malignancies (HMs), in 57 (31%) hematopoietic cell transplantation (HCT) recipients, and in 34 patients (19%) with solid tumors. - Most patients (92%) had a community-acquired infection and presented with upper respiratory tract infections (URTIs) (67%), and 43% developed LRTIs (59 presented with LRTIs and 19 progressed from an URTI to an LRTI) - All-cause mortality at 30 days from HMPV diagnosis was low (4%), and patients with LRTIs had a 10% mortality rate at day 30 from diagnosis, whereas patients with URTIs had a 0% mortality rate |
| Fall 2022^41^ | 2011-2020 | - Systematic literature review to map case fatality rate and prevalence of current and past EV-D68 infections. - Determined prevalence using model random effect | - In 2018, 96% of identified AFM cases in the US were admitted to hospital, 58% to an ICU. |
| Ferreira-Coimbra 2020^42^ | 2000-2019 | - Review, methodology not stated | - In adults under 65 years old, CAP incidence varies between 24.8/10,000 person-years and 106/10,000 person-years. - Elderly people have a higher incidence, representing 63.0/ 10,000 person-years in 65–79-year-olds and reaching 164.3/10,000 person-years after 80 years old. - CAP causes around 102,000 deaths per year, a mortality of 13%, 23.4% and 30.6% at 1 month, 6 months and 12 months, respectively |
| Kim 2022^43^ | 2010-2020 | - Rapid review - Determined rates of hospitalization and/or mortality associated with laboratory-confirmed influenza | - Crude hospitalization rates for lab-confirmed influenza among adults aged 50–64 years ranged from 8.1 (2011–2012) to 112.8 (2017–2018) per 100,000, with an overall average of 56.7 per 100,000 for the 2010–2011 to 2019–2020 seasons. - 5-year average rate of hospitalization using population-based surveillance for 2010–2011 through 2014–2015 was 35.3 per 100,000 compared with a rate of 77.8 per 100,000 for 2015–2016 to 2019–2020. - A population-based study of residents of 14 states estimated influenza-associated hospitalization rate to be 21.9 per 100,000 for 2010–2011; after adjusting lab-confirmed rate for under testing and sensitivity and specificity of lab methods used, estimate increased to 86.1 per 100,000.24 - In surveillance studies, 247 of 7981 (3.1%) adults aged 50–64 years hospitalized with lab-confirmed influenza died during the 2011–2012 through 2014–2015 seasons. |
| Lucero-Obusan 2018^44^ | 2010-2016 | - Surveillance report of influenza activity in the US Veterans Affairs population compared to national CDC FluView data | - Veterans Affairs-confirmed influenza cases ranged from 1005 to 11 506 per season; triage calls from 6090 to 10 346; outpatient visits from 3849 to 13 406; antiviral prescriptions from 3650 to 32 826; hospitalizations from 546 to 4673; and deaths in hospitalized patients from 17 to 139. |
| McLaughlin 2015^45^ | 2011 | - Incidence rates constructed using number of CAP episodes in VHA in   2011 and number of person-years (PYs) accrued in VHA-enrolled population by age and risk groups.  Incidence rate ratios and 95 % confidence  intervals constructed by comparing IRs between various age and risk groups | - In 2011, 34,101 Veterans developed CAP (35,380 episodes) over 7,739,757 VHA person-years. - CAP incidence rates were higher for those aged ≥50 years. - Most Veterans aged 50–64 (53 %) and ≥65 (66 %) years had ≥1 chronic medical (moderate risk) or immunocompromising (high risk) condition. Compared to those at low risk (healthy), moderate- and high-risk Veterans were >3 and >6 times more likely to develop CAP, respectively. - Percentage of CAP patients who were hospitalized was 45%, ranging from 12% (age 18–49, low risk) to 57% (age ≥65, high risk). - One-year all-cause mortality rates ranged from 1% (age 18–49, low risk) to 36% (age ≥65, high risk). - Annual VHA medical expenditure related to CAP estimated to be $750 million ($415M for those aged ≥65 years). |
| Olasupo 2018^46^ | 2014 | - Retrospective analysis to evaluate length of stay, inpatient mortality, 30-day readmissions, and costs of CAP compared to diabetes mellitus, myocardial infarction, and stroke | - 275,790 hospitalizations were analyzed and represented a national estimate of 616,300 hospitalizations, including 269,961 for CAP - Mean LOS was 5.2 days - Median costs were $7282 - 30-day readmission rate was 17% |
| Ortiz 2014^47^ | 2003-2009 | - Modelling study to identify outcomes of interest by ICD-9-CM code and critical illness - Linked hospitalization datasets with virus surveillance datasets by geographic region and month of hospitalization. - Used negative binomial regression models to estimate number of influenza-associated events for the outcomes of interest. | - 80,834 influenza-associated respiratory and circulatory hospitalizations and 26,760 influenza-associated critical illness hospitalizations. - When a pneumonia diagnosis was excluded, estimated number of influenza-associated respiratory and circulatory hospitalizations was 24,816 - Estimated number of influenza-associated critical illness hospitalizations was 8,213 |
| **Combined population** | | | |
| Amand 2018^48^ | 2012-2013 | - Retrospective case-control study - Patients matched 1:1 with non-RSV controls for age, gender, region, healthcare plan and index date. - Stratified analyses for healthcare resource use and costs conducted by age groups. - RSV-attributable resource use and costs assessed based on incremental differences between RSV cases and controls using multivariate analysis | - RSV patients had higher healthcare resource use (hospital stays, ED/urgent care visits, ambulatory visits, outpatient visits) than non-RSV matched controls for all age groups, particularly elderly age groups with RSV (1.9 to 3 days LOS, 0.4 to 0.5 more ED/urgent care visits, 0.7 to 2.7 more ambulatory visits, 12.1 to 18.6 more outpatient visits, 9.5 to 14.6 more prescriptions than elderly in control groups). - Incremental difference in adjusted mean annual costs between RSV and non-RSV controls was higher in elderly (≥65; $12,030 to $23,194) than in those aged <65 years ($2251 to $5391). - Among children, adjusted costs attributable to RSV were higher in children aged 5–17 years ($3192), than in those 1–4 years ($2251 to $2521). |
| Chu 2021^49^ | 2020 | - Examined burden of disease from COVID-19 using attributable mortality | - Across all ages and race/ethnicity groups, attributable mortality from COVID-19 overall was 9.2%. - Rates among Asians, Latinx, Blacks, American Indian/Alaska Native, and Native Hawaiian Pacific Islander were higher than that of non- Hispanic Whites. - Asians had statistically higher attributable mortality compared to non-Hispanic Whites between ages 45 and older. Native Hawaiian Pacific Islanders had the highest attributable mortality between ages 15 and 24, and had higher rates than non-Hispanic Whites between ages 45 and 84. American Indian/Alaska Native s had higher rates than non-Hispanic Whites between ages 45 and 84. Blacks had higher rates than non-Hispanic Whites for those ages 35 and older. Latinx had higher attributable mortality rates than non-Hispanic Whites from age 15 across all ages. |
| Goldstein 2015^50^ | 2003-2011 | - Weekly hospitalization rates for several principal diagnoses by age group were regressed linearly against incidence proxies for major influenza subtypes and RSV adjusting for temporal trends and seasonal baselines | - Highest rate of influenza-associated respiratory hospitalizations was among ≥75 year-olds. - For RSV, highest estimate was in children age <1 - rate was an order of magnitude higher than the corresponding one for influenza. - Significant burden of RSV-associated respiratory hospitalizations found among children 1–4 (average rate of RSV-associated hospitalizations estimated to be 3.2 times as high as corresponding rate for influenza-associated hospitalizations) as well as for ages ≥75. - Most influenza-associated respiratory hospitalizations among children were P&I hospitalizations - Rate of RSV-associated pneumonia and influenza hospitalizations is 1.61 times as high as the rate of influenza-associated pneumonia and influenza hospitalizations for age <1 and 3.13 times as high for ages 1–4. - 51.2% of respiratory RSV-associated hospitalizations for ages ≥75 were hospitalizations for chronic lower respiratory disease (with corresponding share for influenza at 30.2%). |
| Matias 2016^51^ | 2006-2009 | - Extracted weekly time series of visits due to respiratory diagnoses, otitis media, and urinary tract infections. - Used multiple linear regression modelling to estimate age-specific influenza-related excess in office visits. | - Average of ~14.5 M ([SD] across seasons 3.9 million) office visits to influenza (rate of 5,581/100,000 population). - ~80 % of visits occurred in the 5–17 and 18–49 age group. - In school children aged 5–17 years and adult 18–64 years, majority of visits due to influenza B, while A/H3N2 explained most visits in children <5 years. - ~2.2 M otitis media (OM) visits (SD across seasons 790,000) annually to influenza, of which 86% occurred in children <18 years - 6.4% of all infants <2 years and 4.9% of all toddlers aged 2–4 years in the US have an influenza-attributable outpatient visit with an OM diagnosis. - In seniors 65 years and older, ~0.7 M (SD across seasons 351,000) respiratory visits for influenza (rate of 1,887/100,000 population). |
| Near 2022^52^ | 2014-2019 | - Retrospective cohort study - Identified patients with evidence of medically-attended influenza during influenza seasons - Used multivariable logistic regression model to identify patient characteristics that predicted 30-day influenza-related hospitalization. - Selected cohorts of influenza patients and used 1:1 propensity score matching to patients without influenza with similar high-risk characteristics to compare influenza-attributable rates of all-cause hospital and emergency department visits during follow-up. | - From October 2014 to May 2018, more than 1.6 million influenza cases were identified, of which 18,509 (1.2%) had a hospitalization. - Elderly age was associated with 9 times the odds of hospitalization (≥65 years vs. 5–17 years and select comorbidities were associated with 2–3 times the odds of hospitalization. - From October 2014 to March 2019, elderly influenza patients with comorbidities had 3 to 7 times higher 30-day hospitalization rates compared to matched patients without influenza |
| Nguyen 2013^53^ | 2009-2010 | - Age and sex-specific death rates, and age-standardized death rates calculated. - Used negative binomial Serfling-type methods to calculate excess mortality by sex and age groups. | - Compared to typical pattern of seasonal flu deaths, the 2009 pandemic age-specific mortality, as well as influenza-attributable (excess) mortality, skewed much younger. - Estimated 2,634 excess pneumonia and influenza deaths in 2009–10 - Excess death rate in 2009 was 0.79 per 100,000. |
| Palekar 2019^54^ | 2010-2015 | - Estimated influenza-associated hospitalizations rate using monthly number of respiratory hospitalizations, monthly proportion of influenza-positive samples, and census rates. - Used random effects meta-analyses to pool age-group specific rates and extrapolated to countries that did not contribute data, using pooled rates stratified by age group and country characteristics associated with rates | - Burden of influenza-associated respiratory hospitalizations from 2010-2013, per 100,000 people   - Aged <5 years     - Crude range, all respiratory hospitalizations 1,222–1,601     - Crude range, influenza-associated hospitalizations 49–144   - Aged 5–64 years     - Crude range, all respiratory hospitalizations 1,135–1,303     - Crude range, influenza-associated hospitalizations 46–110   - Aged >65 years     - Crude range, all respiratory hospitalizations 8,727–9,900     - Crude range, influenza-associated hospitalizations 287–734 |
| Reed 2015^55^ | 2010-2013 | - Used multiplier method with routine population-based surveillance data on influenza hospitalization to correct for under-reporting and estimate burden of influenza | - Influenza-related hospitalizations were under-detected during 2010-11 by a factor of 2.1 for age < 18 years, 3.1 for ages 18-64 years, and 5.2 for age 65+. Results were similar in 2011-12. - Extrapolated estimates for 3 seasons from 2010–2013 included 114,192–624,435 hospitalizations, 18,491–95,390 ICU admissions, and 4,915–27,174 deaths per year - 54–70% of hospitalizations and 71–85% of deaths occurred among adults aged 65+. |
| Rolfes 2018^56^ | 2010-2011; 2015-2016 | - Used routinely collected surveillance data, outbreak field investigations, and proportions of people seeking health care from survey results to estimate number of illnesses, medical visits, hospitalizations, and deaths due to influenza | - Over the past six influenza seasons (2010-2011 through 2015-2016), influenza-associated illnesses estimates ranged from a low of 9.2 million to a high of 35.6 million illnesses, with variation by age. - Outpatient medical visits related to influenza ranged from 4.3 million to 16.7 million - Influenza-associated hospitalizations ranged from 139 000 to 708 000. - From 2010-2011 through 2013-2014 influenza seasons, influenza-associated respiratory and circulatory deaths ranged from a low of 12 000 to a high of 56 000, and associated pneumonia and influenza deaths ranged from 4000 to 12 000 over the six seasons from 2010-2011 through 2015-2016. |
| Sibbel 2016^57^ | 2009-2011 | - Used linear mixed-effects models to assess hospitalization rates and costs over 3 months prior to and 12 months following pneumonia episodes | - Pneumonia incidence rate for 2009 to 2011 was 21.4 events/100 patient-years; most episodes (90.1%) required inpatient treatment. - 30-day case fatality rate was 10.7%. - Compared to month -3 prior to event, rates of all-cause and cardiovascular hospitalization were higher in the month of the pneumonia episode - All-cause admission rates remained elevated through month 12; cardiovascular admission rates remained elevated through month 6. - Mean per-patient per-month costs were $10,976 higher in the month of index episode compared to month -3, largely driven by increased inpatient costs, and remained elevated through end of 12-month follow-up. |
| Tsai 2014^58^ | 2007-2009 | - Calculated mean work-loss hours per ILI episode and proportion of employees with at least one ILI episode. Work-loss hours and ILI rates were examined by subgroups | - The mean number of work hours lost per ILI episode was 23.6 in 2007–8 and 23.9 in 2008–9. - The proportion of employees with at least one ILI was 1.7% in 2007–8 and 1.2% in 2008–9. - In both seasons, the proportion with ILI was higher among older (2.1 and 1.5%) and hourly workers (2.0 and 1.3%), workers in the southern region (1.9 and 1.3%) and those in oil, gas, or mining industries (1.9 and 1.4%). |
| Wroe 2012^59^ | estimates for 2004,  2010, 2020, 2030, and 2040 from US Census Bureau projections | - Using decision tree–based probabilistic model to estimate impact of population growth and projected future shifts in age distribution of the US population on national burden of pneumococcal pneumonia. - Applied 2004 rates of pneumonia to future population projections to assess potential changes in number of episodes of overall pneumonia and pneumococcal pneumonia, and associated healthcare utilization, outcomes, and costs due to pneumococcal pneumonia in coming decades | - Overall U.S. population is expected to increase by 38%, from 294 million to 406 million, between 2004 and 2040. - The elderly (≥65 years) population will grow from 36 million to 81 million, and the oldest old (≥85 years) population will nearly triple, from 5 million to 14 million. - All-cause outpatient pneumonia - estimated increase from 2.5 million episodes in 2004 to 3.5 million episodes in 2040. - Pneumococcal pneumonia - estimated 490 000 cases of outpatient pneumococcal pneumonia in 2004; 520 000 in 2010; 580 000 in 2020; 640 000 in 2030; and 700 000 in 2040. - The fraction of outpatient pneumonia accounted for by the elderly will increase from 15% in 2004 to 23% in 2040 - The fraction of outpatient pneumonia accounted for by the oldest old will increase from 2% to 4%. - Hospitalizations due to all-cause pneumonia are projected to increase by nearly 100%, from 1.3 million to 2.6 million, between 2004 and 2040. - In 2007 dollars, total direct costs are predicted to double from $2.5 billion in 2004 to $5.0 billion in 2040, with the largest proportional increase in costs between 2020 and 2030 (25% increase from $3.3 billion to $4.2 billion). The majority (93%–95%) of direct costs in all years studied were due to inpatient episodes. |
| Young-Xu 2017^60^ | 2010-2014 | - Estimated influenza-attributed outcomes using statistical regression model of observed emergency department visits, hospitalizations, and deaths from electronic medical records and respiratory viral surveillance data | - An estimated 10,674 VA ED visits, 2,538 VA hospitalizations, 5,522 all-cause deaths, and 3,793 underlying respiratory or circulatory deaths (inside and outside VA) among adult Veterans were attributable to influenza each year from 2010 through 2014. - Annual value of lost productivity amounted to $27 million, annual costs for ED visits were $6.2 million. - 96% of VA hospitalizations resulted in either death or a discharge to home, with annual costs totaling $36 million. The remaining 4% of hospitalizations were followed by extended care at rehabilitation and skilled nursing facilities with annual costs totaling $5.5 million. - The annual monetary value of quality-adjusted life years (QALYs) lost amounted to $1.1 billion. |

# References

1. Antoon JW, Hall M, Herndon A, et al. Prevalence, Risk Factors, and Outcomes of Influenza-Associated Neurologic Complications in Children. *J Pediatr*. Dec 2021;239:32-38.e5. doi:10.1016/j.jpeds.2021.06.075

2. Antoon JW, Hall M, Howard LM, et al. COVID-19 and Acute Neurologic Complications in Children. *Pediatrics*. Nov 1 2022;150(5)doi:10.1542/peds.2022-058167

3. Danziger-Isakov L, Steinbach WJ, Paulsen G, et al. A Multicenter Consortium to Define the Epidemiology and Outcomes of Pediatric Solid Organ Transplant Recipients With Inpatient Respiratory Virus Infection. *J Pediatric Infect Dis Soc*. Jul 1 2019;8(3):197-204. doi:10.1093/jpids/piy024

4. Williams DJ, Zhu Y, Grijalva CG, et al. Predicting Severe Pneumonia Outcomes in Children. *Pediatrics*. Oct 2016;138(4)doi:10.1542/peds.2016-1019

5. Ghimire LV, Chou FS, Moon-Grady AJ. Impact of congenital heart disease on outcomes among pediatric patients hospitalized for influenza infection. *BMC Pediatr*. Sep 28 2020;20(1):450. doi:10.1186/s12887-020-02344-x

6. Leyenaar JK, Lagu T, Shieh MS, Pekow PS, Lindenauer PK. Management and outcomes of pneumonia among children with complex chronic conditions. *Pediatr Infect Dis J*. Sep 2014;33(9):907-11. doi:10.1097/inf.0000000000000317

7. Wilking AN, Elliott E, Garcia MN, Murray KO, Munoz FM. Central nervous system manifestations in pediatric patients with influenza A H1N1 infection during the 2009 pandemic. *Pediatr Neurol*. Sep 2014;51(3):370-6. doi:10.1016/j.pediatrneurol.2014.04.026

8. Zambrano LD, Ly KN, Link-Gelles R, et al. Investigating Health Disparities Associated With Multisystem Inflammatory Syndrome in Children After SARS-CoV-2 Infection. *Pediatr Infect Dis J*. Nov 1 2022;41(11):891-898. doi:10.1097/inf.0000000000003689

9. Carr SB, Adderson EE, Hakim H, Xiong X, Yan X, Caniza M. Clinical and demographic characteristics of seasonal influenza in pediatric patients with cancer. *Pediatr Infect Dis J*. Nov 2012;31(11):e202-7. doi:10.1097/INF.0b013e318267f7d9

10. Chaves SS, Perez A, Farley MM, et al. The burden of influenza hospitalizations in infants from 2003 to 2012, United States. *Pediatr Infect Dis J*. Sep 2014;33(9):912-9. doi:10.1097/inf.0000000000000321

11. Bailey KL, Sayles H, Campbell J, et al. COVID-19 patients with documented alcohol use disorder or alcohol-related complications are more likely to be hospitalized and have higher all-cause mortality. *Alcohol Clin Exp Res*. Jun 2022;46(6):1023-1035. doi:10.1111/acer.14838

12. Hussein MH, Toraih EA, Attia AS, et al. Asthma in COVID-19 patients: An extra chain fitting around the neck? *Respir Med*. Dec 2020;175:106205. doi:10.1016/j.rmed.2020.106205

13. Fox T, Ruddiman K, Lo KB, et al. The relationship between diabetes and clinical outcomes in COVID-19: a single-center retrospective analysis. *Acta Diabetol*. Jan 2021;58(1):33-38. doi:10.1007/s00592-020-01592-8

14. Melmed KR, Cao M, Dogra S, et al. Risk factors for intracerebral hemorrhage in patients with COVID-19. *J Thromb Thrombolysis*. May 2021;51(4):953-960. doi:10.1007/s11239-020-02288-0

15. Page-Wilson G, Arakawa R, Nemeth S, et al. Obesity is independently associated with septic shock, renal complications, and mortality in a multiracial patient cohort hospitalized with COVID-19. *PLoS One*. 2021;16(8):e0255811. doi:10.1371/journal.pone.0255811

16. Panhwar MS, Kalra A, Gupta T, et al. Relation of Concomitant Heart Failure to Outcomes in Patients Hospitalized With Influenza. *Am J Cardiol*. May 1 2019;123(9):1478-1480. doi:10.1016/j.amjcard.2019.01.046

17. Ponce J, Anzalone AJ, Bailey K, et al. Impact of malnutrition on clinical outcomes in patients diagnosed with COVID-19. *JPEN J Parenter Enteral Nutr*. Nov 2022;46(8):1797-1807. doi:10.1002/jpen.2418

18. Shah NS, Greenberg JA, McNulty MC, et al. Severe Influenza in 33 US Hospitals, 2013-2014: Complications and Risk Factors for Death in 507 Patients. *Infect Control Hosp Epidemiol*. Nov 2015;36(11):1251-60. doi:10.1017/ice.2015.170

19. Wyffels V, Kariburyo F, Gavart S, Fleischhackl R, Yuce H. A Real-World Analysis of Patient Characteristics and Predictors of Hospitalization Among US Medicare Beneficiaries with Respiratory Syncytial Virus Infection. *Adv Ther*. Mar 2020;37(3):1203-1217. doi:10.1007/s12325-020-01230-3

20. Placzek HE, Madoff LC. Association of age and comorbidity on 2009 influenza A pandemic H1N1-related intensive care unit stay in Massachusetts. *Am J Public Health*. Nov 2014;104(11):e118-25. doi:10.2105/ajph.2014.302197

21. Arriola CS, Kim L, Langley G, et al. Estimated Burden of Community-Onset Respiratory Syncytial Virus-Associated Hospitalizations Among Children Aged <2 Years in the United States, 2014-15. *J Pediatric Infect Dis Soc*. Nov 10 2020;9(5):587-595. doi:10.1093/jpids/piz087

22. Chirikov VV, Simoes EAF, Kuznik A, Kwon Y, Botteman M. Economic-Burden Trajectories in Commercially Insured US Infants With Respiratory Syncytial Virus Infection. *J Infect Dis*. Mar 28 2020;221(8):1244-1255. doi:10.1093/infdis/jiz160

23. Edwards KM, Zhu Y, Griffin MR, et al. Burden of human metapneumovirus infection in young children. *N Engl J Med*. Feb 14 2013;368(7):633-43. doi:10.1056/NEJMoa1204630

24. Fell DB, Johnson J, Mor Z, et al. Incidence of laboratory-confirmed influenza disease among infants under 6 months of age: a systematic review. *BMJ Open*. Sep 7 2017;7(9):e016526. doi:10.1136/bmjopen-2017-016526

25. Fletcher MA, Schmitt HJ, Syrochkina M, Sylvester G. Pneumococcal empyema and complicated pneumonias: global trends in incidence, prevalence, and serotype epidemiology. *Eur J Clin Microbiol Infect Dis*. Jun 2014;33(6):879-910. doi:10.1007/s10096-014-2062-6

26. Kurup S, Burgess R, Tine F, Chahroudi A, Lee DL. SARS-CoV-2 Infection and Racial Disparities in Children: Protective Mechanisms and Severe Complications Related to MIS-C. *J Racial Ethn Health Disparities*. Aug 2022;9(4):1536-1542. doi:10.1007/s40615-021-01092-7

27. Ma KC, Winn A, Moline HL, et al. Increase in Acute Respiratory Illnesses Among Children and Adolescents Associated with Rhinoviruses and Enteroviruses, Including Enterovirus D68 - United States, July-September 2022. *MMWR Morb Mortal Wkly Rep*. Oct 7 2022;71(40):1265-1270. doi:10.15585/mmwr.mm7140e1

28. McLaughlin JM, Khan F, Schmitt HJ, et al. Respiratory Syncytial Virus-Associated Hospitalization Rates among US Infants: A Systematic Review and Meta-Analysis. *J Infect Dis*. Mar 15 2022;225(6):1100-1111. doi:10.1093/infdis/jiaa752

29. Simoes EAF. The Burden of Respiratory Syncytial Virus Lower Respiratory Tract Disease in Infants in the United States: A Synthesis. *J Infect Dis*. Aug 15 2022;226(Suppl 2):S143-S147. doi:10.1093/infdis/jiac211

30. Suh M, Movva N, Bylsma LC, Fryzek JP, Nelson CB. A Systematic Literature Review of the Burden of Respiratory Syncytial Virus and Health Care Utilization Among United States Infants Younger Than 1 Year. *J Infect Dis*. Aug 15 2022;226(Suppl 2):S195-s212. doi:10.1093/infdis/jiac201

31. Suh M, Movva N, Jiang X, et al. Respiratory Syncytial Virus Burden and Healthcare Utilization in United States Infants <1 Year of Age: Study of Nationally Representative Databases, 2011-2019. *J Infect Dis*. Aug 15 2022;226(Suppl 2):S184-S194. doi:10.1093/infdis/jiac155

32. Bolge SC, Gutierrez C, Kariburyo F, He D. Burden of Pneumonia Among Hospitalized Patients with Influenza: Real-World Evidence from a US Managed Care Population. *Pulm Ther*. Dec 2021;7(2):517-532. doi:10.1007/s41030-021-00169-2

33. Brown JD, Harnett J, Chambers R, Sato R. The relative burden of community-acquired pneumonia hospitalizations in older adults: a retrospective observational study in the United States. *BMC Geriatr*. Apr 16 2018;18(1):92. doi:10.1186/s12877-018-0787-2

34. Cavallazzi R, Furmanek S, Arnold FW, et al. The Burden of Community-Acquired Pneumonia Requiring Admission to ICU in the United States. *Chest*. Sep 2020;158(3):1008-1016. doi:10.1016/j.chest.2020.03.051

35. Charu V, Simonsen L, Lustig R, Steiner C, Viboud C. Mortality burden of the 2009-10 influenza pandemic in the United States: improving the timeliness of influenza severity estimates using inpatient mortality records. *Influenza Other Respir Viruses*. Sep 2013;7(5):863-71. doi:10.1111/irv.12096

36. Datta S, Walsh EE, Peterson DR, Falsey AR. Can Analysis of Routine Viral Testing Provide Accurate Estimates of Respiratory Syncytial Virus Disease Burden in Adults? *J Infect Dis*. Jun 1 2017;215(11):1706-1710. doi:10.1093/infdis/jix196

37. de Courville C, Cadarette SM, Wissinger E, Alvarez FP. The economic burden of influenza among adults aged 18 to 64: A systematic literature review. *Influenza Other Respir Viruses*. May 2022;16(3):376-385. doi:10.1111/irv.12963

38. Drijkoningen JJ, Rohde GG. Pneumococcal infection in adults: burden of disease. *Clin Microbiol Infect*. May 2014;20 Suppl 5:45-51. doi:10.1111/1469-0691.12461

39. Ehrlich HY, Harizaj A, Campbell L, et al. SARS-CoV-2 in Nursing Homes after 3 Months of Serial, Facilitywide Point Prevalence Testing, Connecticut, USA. *Emerg Infect Dis*. May 2021;27(5):1288-1295. doi:10.3201/eid2705.204936

40. El Chaer F, Shah DP, Kmeid J, et al. Burden of human metapneumovirus infections in patients with cancer: Risk factors and outcomes. *Cancer*. Jun 15 2017;123(12):2329-2337. doi:10.1002/cncr.30599

41. Fall A, Kenmoe S, Ebogo-Belobo JT, et al. Global prevalence and case fatality rate of Enterovirus D68 infections, a systematic review and meta-analysis. *PLoS Negl Trop Dis*. Feb 2022;16(2):e0010073. doi:10.1371/journal.pntd.0010073

42. Ferreira-Coimbra J, Sarda C, Rello J. Burden of Community-Acquired Pneumonia and Unmet Clinical Needs. *Adv Ther*. Apr 2020;37(4):1302-1318. doi:10.1007/s12325-020-01248-7

43. Kim DK, McGeer A, Uleryk E, Coleman BL. Burden of severe illness associated with laboratory confirmed influenza in adults aged 50-64 years: A rapid review. *Influenza Other Respir Viruses*. Jul 2022;16(4):632-642. doi:10.1111/irv.12955

44. Lucero-Obusan C, Schirmer PL, Wendelboe A, Oda G, Holodniy M. Epidemiology and burden of influenza in the U.S. Department of Veterans Affairs. *Influenza Other Respir Viruses*. Mar 2018;12(2):293-298. doi:10.1111/irv.12512

45. McLaughlin JM, Johnson MH, Kagan SA, Baer SL. Clinical and economic burden of community-acquired pneumonia in the Veterans Health Administration, 2011: a retrospective cohort study. *Infection*. Dec 2015;43(6):671-80. doi:10.1007/s15010-015-0789-3

46. Olasupo O, Xiao H, Brown JD. Relative Clinical and Cost Burden of Community-Acquired Pneumonia Hospitalizations in Older Adults in the United States-A Cross-Sectional Analysis. *Vaccines (Basel)*. Aug 31 2018;6(3)doi:10.3390/vaccines6030059

47. Ortiz JR, Neuzil KM, Cooke CR, Neradilek MB, Goss CH, Shay DK. Influenza pneumonia surveillance among hospitalized adults may underestimate the burden of severe influenza disease. *PLoS One*. 2014;9(11):e113903. doi:10.1371/journal.pone.0113903

48. Amand C, Tong S, Kieffer A, Kyaw MH. Healthcare resource use and economic burden attributable to respiratory syncytial virus in the United States: a claims database analysis. *BMC Health Serv Res*. Apr 20 2018;18(1):294. doi:10.1186/s12913-018-3066-1

49. Chu JN, Tsoh JY, Ong E, Ponce NA. The Hidden Colors of Coronavirus: the Burden of Attributable COVID-19 Deaths. *J Gen Intern Med*. May 2021;36(5):1463-1465. doi:10.1007/s11606-020-06497-4

50. Goldstein E, Greene SK, Olson DR, Hanage WP, Lipsitch M. Estimating the hospitalization burden associated with influenza and respiratory syncytial virus in New York City, 2003-2011. *Influenza Other Respir Viruses*. Sep 2015;9(5):225-33. doi:10.1111/irv.12325

51. Matias G, Haguinet F, Lustig RL, Edelman L, Chowell G, Taylor RJ. Model estimates of the burden of outpatient visits attributable to influenza in the United States. *BMC Infect Dis*. Nov 7 2016;16(1):641. doi:10.1186/s12879-016-1939-7

52. Near AM, Tse J, Young-Xu Y, Hong DK, Reyes CM. Burden of influenza hospitalization among high-risk groups in the United States. *BMC Health Serv Res*. Sep 28 2022;22(1):1209. doi:10.1186/s12913-022-08586-y

53. Nguyen AM, Noymer A. Influenza mortality in the United States, 2009 pandemic: burden, timing and age distribution. *PLoS One*. 2013;8(5):e64198. doi:10.1371/journal.pone.0064198

54. Palekar RS, Rolfes MA, Arriola CS, et al. Burden of influenza-associated respiratory hospitalizations in the Americas, 2010-2015. *PLoS One*. 2019;14(9):e0221479. doi:10.1371/journal.pone.0221479

55. Reed C, Chaves SS, Daily Kirley P, et al. Estimating influenza disease burden from population-based surveillance data in the United States. *PLoS One*. 2015;10(3):e0118369. doi:10.1371/journal.pone.0118369

56. Rolfes MA, Foppa IM, Garg S, et al. Annual estimates of the burden of seasonal influenza in the United States: A tool for strengthening influenza surveillance and preparedness. *Influenza Other Respir Viruses*. Jan 2018;12(1):132-137. doi:10.1111/irv.12486

57. Sibbel S, Sato R, Hunt A, Turenne W, Brunelli SM. The clinical and economic burden of pneumonia in patients enrolled in Medicare receiving dialysis: a retrospective, observational cohort study. *BMC Nephrol*. Dec 12 2016;17(1):199. doi:10.1186/s12882-016-0412-6

58. Tsai Y, Zhou F, Kim IK. The burden of influenza-like illness in the US workforce. *Occup Med (Lond)*. Jul 2014;64(5):341-7. doi:10.1093/occmed/kqu022

59. Wroe PC, Finkelstein JA, Ray GT, et al. Aging population and future burden of pneumococcal pneumonia in the United States. *J Infect Dis*. May 15 2012;205(10):1589-92. doi:10.1093/infdis/jis240

60. Young-Xu Y, van Aalst R, Russo E, Lee JK, Chit A. The Annual Burden of Seasonal Influenza in the US Veterans Affairs Population. *PLoS One*. 2017;12(1):e0169344. doi:10.1371/journal.pone.0169344
